# Dorsal tongue porphyrin autofluorescence and *Candida* saprophytism: A prospective observational study

**DOI:** 10.1371/journal.pone.0223072

**Published:** 2019-09-26

**Authors:** Massimo Petruzzi, Fedora della Vella, Andrea Cassandro, Adriana Mosca, Mariasevera Di Comite, Maria Contaldo, Felice Roberto Grassi, Dorina Lauritano

**Affiliations:** 1 Interdisciplinary Department of Medicine, University “Aldo Moro” of Bari, Bari, Italy; 2 Department of Basic Medical Sciences, Neurosciences and Sensory Organs, University of Bari “Aldo Moro”, Bari, Italy; 3 Multidisciplinary Department of Medical-Surgical and Odontostomatological Specialties, University of Campania “Luigi Vanvitelli”, Naples, Italy; 4 Department of Medicine and Surgery, University Milano-Bicocca, Monza, Italy; Massachusetts General Hospital, UNITED STATES

## Abstract

**Aim:**

To investigate the correlation between the dorsal tongue porphyrin autofluorescence, revealed using VELscope, and *Candida* saprophytism.

**Material and methods:**

Consecutive patients underwent an autofluorescence examination by the VELscope device to establish the presence or absence of porphyrin fluorescence. A tongue swab was collected for the *Candida* cultural test. Sensitivity, specificity, accuracy, negative predictive value and positive predictive value were calculated considering the oral swab as the gold standard. The degree of agreement between the two tests was calculated using Cohen's K coefficient.

**Results:**

One hundred twenty-six patients were enrolled. Porphyrin fluorescence method showed a sensitivity of 78%, specificity of 76% and an accuracy of 78%. Negative predictive value and positive predictive value were respectively 90% and 59%. The strength of agreement between the two methods resulted to be moderate (k = 0.551).

**Conclusions:**

Off-label use of tongue autofluorescence examination to detect the presence of *Candida* species is characterized by a loss of porphyrin fluorescence. The high negative predictive value of porphyrin fluorescence loss suggests its use in preliminary selection of *Candida* carriers, in order to plan preventive and therapeutic strategies.

## Introduction

Autofluorescence is a biochemical-physical characteristic of the tissues that excited with an appropriate blue light source (400-460-nm), emit light with a higher wavelength [1]. Endogenous fluorophores as keratin, collagen, elastin, NADH and porphyrins are the molecules responsible of this phenomenon. On the other hand, haemoglobin and melanin tends to absorb the blue incident light, reducing the tissue autofluorescence [2]. Tissue modifications induced by neoplastic, potentially malignant, inflammatory and infective processes can affect the normal tissue autofluorescence pattern; actually, on this principle is based the use of autofluorescence for the early detection of upper aero-digestive tract cancers and related potentially malignant disorders [3–5]. McAlpine et al. classified four different patterns of autofluorescence according to their appearance during the inspection. In particular, normal healthy tissue emits pale green autofluorescence, differently, loss of fluorescence (LOF), characterized by a dark brown to black mucosal area, is typical of dysplastic and/or neoplastic tissues. Gained fluorescence (GF) is usually observed in hyperkeratotic lesions (leukoplakia or *morsicatio buccarum*), while a porphyrin fluorescence (PF) is characterized by a red/orange light emission, often observed on the dorsal tongue surface. The PF is characterized by a 636 nm peaks of emission caused by porphyrins produced by micro-organisms colonizing the dorsal tongue surface [6]. The soft and hard palate can also show a PF, due to their contact with the tongue [7].

The microscopic analysis conducted by Hamad et al. revealed high numbers of bacteria such as *Streptococcus salivarius*, *Actinomyces spp*. and lactobacilli on the mucosal sites emitting a PF, confuting the hypothesis that the PF found in oral squamous cell carcinomas originates from cellular enzymatic synthesis defects [8].

To date, it has not yet been proved if *Candida* species are somehow involved in oral PF, since *Candida* species are usually commensal micro-organisms found in the oral cavity in the 40% of healthy population [9]. The frequency of isolation of *C*. *albicans* and its mucosal density per unit area, as measured by imprint culture, is highest on the dorsum of the tongue, particularly the posterior half. For this reason, tongue is considered the primary oral reservoir of the fungus, from which the rest of the oral mucosa, plaque-coated surfaces of the teeth and the saliva may become secondarily colonized [10].

*Candida* is currently detectable in the oral cavity by swab, smear or oral rinses, followed by a specific microbial culture. Its colonization of the oral tissues can alter the normal microbiota balance, eventually resulting in autofluorescence properties modification.

Aim of this study was to test the efficacy of the off-label autofluorescence tongue examination for *Candida* colonization detection.

## Material and methods

### Study design

This single-centre, prospective, single-arm, observational study carried out at Oral Medicine Section of Dental Clinic of University of Bari (Bari, Italy) from January 2018 to December 2018, was conducted in accordance with the Declaration of Helsinki and independently approved and reviewed by the Institution’s Ethical Committee (Comitato Etico del Policlinico di Bari, prot. n. 46815/C.E.).

### Patients

Consecutive healthy subjects aged between 9 and 80 years, referred to the Dental Clinic of University “Aldo Moro” of Bari, were enrolled. The sample size calculation was done referring to an estimate population of Italians aged between 9 to 80 years, 40% of *Candida* saprophytism prevalence, using n = N^x^/[(N-1)E^2^+x], with 96% confidence level and 9% margin of error.

Written informed consent was obtained from each subject or /and from the parents/guardians of underaged patients attending the study. Individuals were excluded if assumed antimycotic and/or antibiotic drugs in the past 30 days, daily brushed their tongue or refused to give the consent to the study. For each subject demographic data were collected.

### Autofluorescence examination

**Fig 1:**
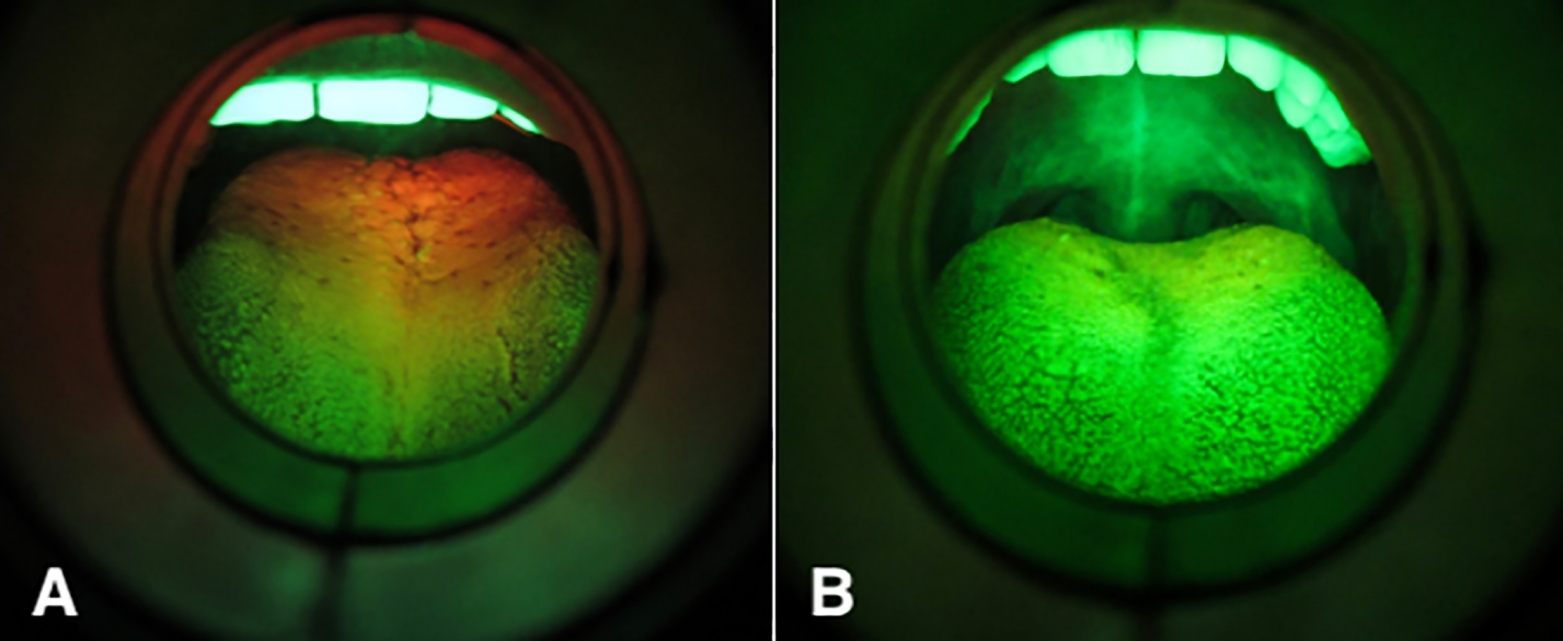
Autofluorescence pictures. (A) Tongue presenting with porphyrin autofluorescence. (B) Tongue not presenting with porphyrin autofluorescence.

The dorsal tongue autofluorescence investigation was performed in all subjects using the medical device VELscope Vx (Mectron s.p.a—Carasco, Genova). The procedure was performed in a shaded room, keeping the device 5 cm distant from the tongue, pulled out by a second operator with a gauze. The excitation blue light (excitation wavelength between 400 and 460 nm) was projected on the dorsal tongue mucosa by a dichroic mirror. Through the back of the handpiece, tissue autofluorescence was evaluated and classified as positive to PF if its aspect was red or orange, on the contrary it was classified as negative to PF (Fig 1). Four experienced experts in oral medicine, and routinely users of VELscope device, previously calibrated, rated as PF positive or negative each single case: only subjects showing an evident and generalized PF were considered positive. The inter-rater reliability was guaranteed setting a percent agreement goal of 95%.

Each case was photographed in autofluorescence, using Canon Powershot Elph 130 IS (Canon Inc, Tokyo, Japan) with a dedicated adapter provided by the manufacturer.

### Oral Swab

Every subject underwent to an oral swab of the dorsal tongue after the VELscope exam. The swab was performed using a sterilized cotton swab (2150/SG, Nuova APTACA S.r.l., Canelli -AT-, Italy), rubbed and rotated on the whole surface of the dorsal tongue. In case of PF positivity, the swab was performed on the red/orange revealed area, in order to avoid inter-areas contamination. The collected specimens were put in a sterile propylene cup and then sown on a solid ground, the Sabouraud Agar Destrosio agar. The culture test revealed the eventual presence of *Candida* and the species involved.

### Data analysis and statistics

The χ^2^ test (significance set at p < 0.05) was used to test oral swab and PF outcomes (positive or negative) in relation to the sample gender and age. Global validation of the PF test results was established by calculating the sensitivity, specificity, accuracy and both the positive and negative predictive values from contingency tables.

The Cohen’s kappa coefficient was used to evaluate the interrater agreement between oral swab and PF. Concordance was evaluated according to Landis and Koch who defined values <0 as indicating no agreement, 0 to 0.20 as slight, 0.21 to 0.40 as fair, 0.41 to 0.60 as moderate, 0.61 to 0.80 as substantial, and 0.81 to 1 as perfect agreement [11].

**Fig 2:**
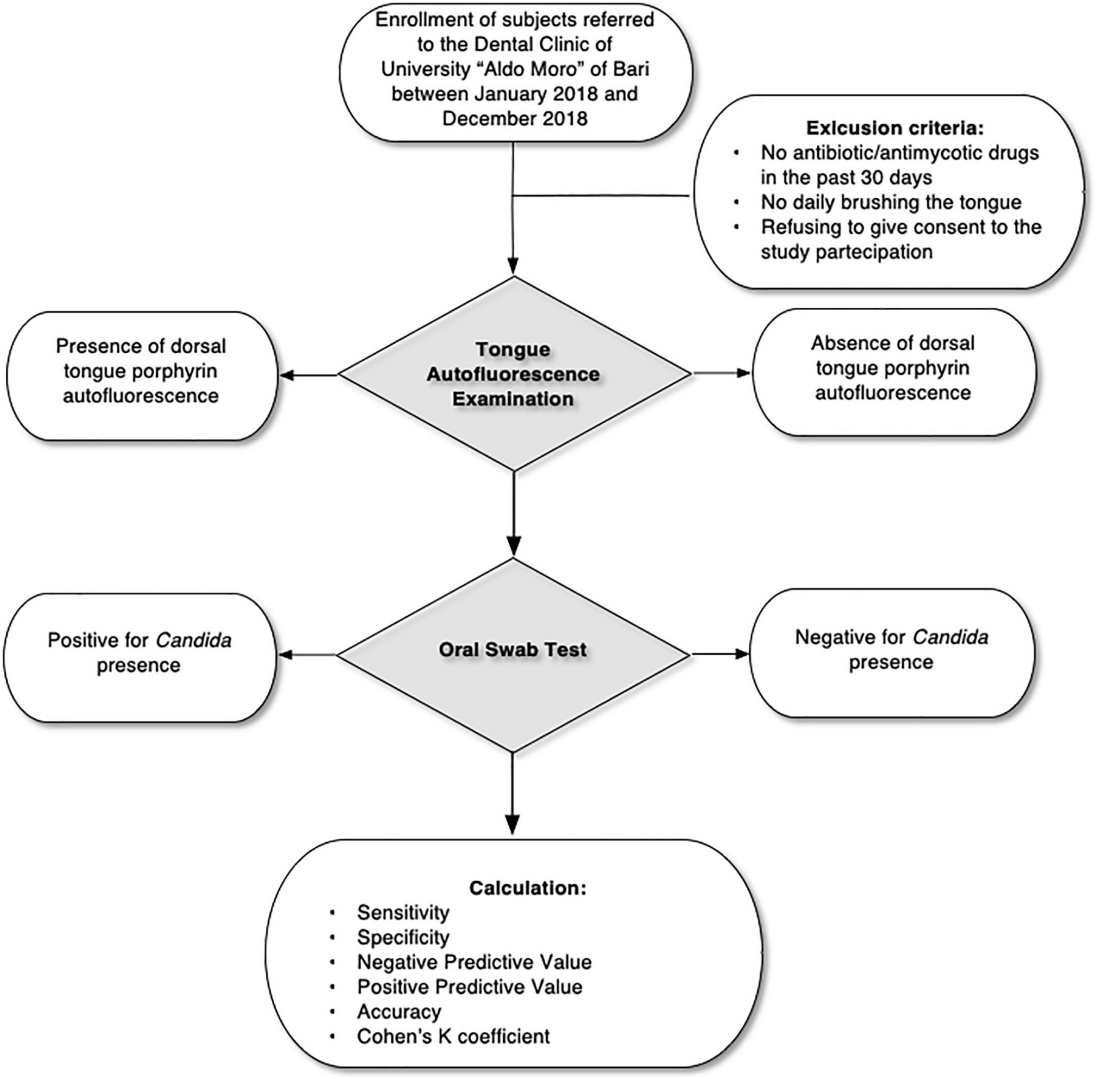
Flow-chart. Study protocol flow-chart.

The study flow-chart is resumed in Fig 2.

## Results

One hundred twenty-six subjects were enrolled, including 51 males and 75 females. The mean age was 55 ± 17 years (mean±SD). Seventy-seven (61%) showed PF at the autofluorescence evaluation, and 37 (29%) participants resulted with a positive culture test for fungi of *Candida* species.

Out of the 49 subjects without PF, 29 were affected by *Candida* colonization, while out of the 77 subjects with PF, only 8 resulted positive to the culture test. The inter-raters’ agreement was 100%.

PF and swab outcomes were not statistically influenced by age and sex (p>0.05).

PF method showed a sensitivity of 78% and specificity of 76% in detecting *Candida* presence.

PPV and NPV were respectively 59% and 90%, with an autofluorescence accuracy rate of 78%. The two methods showed a moderate level of agreement, according to Cohen’s k coefficient (k = 0.551).

The above-mentioned data are resumed in Table 1.

**Table 1:** Demographic and statistical data of the study.

	Total (%)	Swab + (%)	Swab—(%)		p value	PF+ (%)		PF- (%)	p value
**Men**	51 (40)	17 (33)	34 (67)		**p>0.05**	28(55)		23 (45)	**p>0.05**
**Women**	75 (60)	20 (27)	55 (73)			50 (70)		26 (30)	
**Aged<55 yrs**	59 (46)	18 (31)	41 (69)		**p>0.05**	32 (54)		27 (56)	**p>0.05**
**Aged>55 yrs**	67 (54)	19 (28)	48 (72)			45 (67)		22 (33)	
**AUTOFLUORESCENCE**				**ORAL SWAB**					
				*Positive for Candida*			*Negative for Candida*		
*Absence of porphyrin fluorescence*				29 (True Positive)			20 (False Positive)		
*Presence of porphyrin fluorescence*				8 (False Negative)			69 (True Negative)		
				**Value**			**95% C.I.**		
**Sensitivity**				78%			61.9% to 90.17%		
**Specificity**				76%			67.45% to 85.70%		
**Negative Predictive Value**				90%			82.22% to 94.15%		
**Positive Predictive Value**				59%			48.75% to 68.85%		
**Accuracy**				78%			69.51% to 84.70%		
**Cohen’s K coefficient**				0.551			0.356 to 0.665		
**Strength of agreement**				Moderate					

## Discussion

In this study the correlation between *Candida* presence on the dorsal tongue and PF elicited by an off-label use of VELscope was investigated. The method displayed moderate values of sensitivity and specificity (78% and 76%) compared to swab specimens culture test, which remains the gold standard to detect *Candida* species and to perform an antimycogram to start an effective therapy in case of candidiasis. Nevertheless, the high negative predictive value of PF (90%) make it a useful and immediate chair side, non-invasive screening tool, that permit to select the patients needing further microbial investigations.

Conventionally, autofluorescence is an ancillary tool for oral cancer and dysplastic lesions examination, with sensitivity and specificity values of 76% and 66.29% respectively, and NPV and PPV of 95.08% and 24.36% [12] but some off-label uses have also been suggested, like the valuation of the bone vitality status after surgical treatment of Medication Related Osteonecrosis of the Jaws [13].

Fungal optical fluorescence properties have been deeply studied, even if especially in vitro: Rao S. et al. reported about the use of autofluorescence techniques as a rapid screening method for identification of fungi on routinely prepared hematoxylin-eosin-stained tissue sections slides. They found sensitivity and specificity of 97.8% and 100% respectively, concluding that autofluorescence does not require any other specialized staining procedure for fungi detection [14]. The validity of fluorescence microscopy to diagnose *Candida* infection is also well-established [15], providing a sensitive and specific screening tool, compared to the current gold standard, i.e. periodic acid Schiff (PAS) stain [16]. Gabrielli et al. compared in mice the bioluminescence in vivo imaging technique with colony forming units (CFU) measurement for the *Candida* detection. They concluded that the bioluminescence technique was more reliable than CFU counts in detecting mouse’s early oral candidiasis [17]. The typical fluorescence aspect of hyphae is a yellowish-green signal, due to their flavin content [16].

Red autofluorescence, instead, is a common find of the oral cavity, generally localized on the dorsal tongue, and has been historically ascribed to multiple etiopathological theories.

Initial data reported in Literature, attributed PF to dysplastic and/or neoplastic oral tissues derailment [18] but successive assessments defined the correlation between LOF and neoplastic changes [1].

Otherwise, other authors considered the dorsal tongue PF as the physiological status, since it was commonly recorded using the Wood’s lamp in apparently healthy population and assumed that a reduction of PF involved nutritional deficiency and pathological conditions, such as tropical sprue, iron deficiency and pernicious anaemia [19, 20].

Carrie, in 1934, argued that the normal fluorescence of the tongue observed with Wood's lamp was caused by specific Gram-positive bacilli. The same author tried to cultivate these bacilli but did not obtain satisfactory results [21].

Tomaszewski [22] studied the effects of penicillin on PF. After four to five days of oral or inhaled treatment, the PF began to disappear, starting from the anterior area and proceeding towards the back of the base of the tongue. At the end of the therapy, the red fluorescence gradually reappeared, starting from the back of the tongue. Intramuscular penicillin administration did not produce loss of PF.

The hypothesis of a bacterial origin of PF is supported by different studies assessing the emission of red autofluorescence by oral biofilm [23, 24]. Also, halitosis, known to be mainly caused by bacteria, has been linked to porphyrin fluorescence as demonstrated by Lee et al. [25] and Hitz Lindenmüller et al. [26].

Nevertheless, the pathway of porphyrin production is still unknown, if directly synthesized by the bacteria or haemoglobin disruption product. PF has also been suggested as real time detection method for oropharynx bacterial infection [27].

PF was also found in the nasal vestibule, the labial commissure and the vaginal mucosa, especially in the menstrual phase, probably because of the porphyrin produced from the bacterial decomposition of the blood [28]. All the above-mentioned anatomical areas own a typical microbiota, whom alterations could produce a loss of PF. *Candida* colonization, not uncommon on these mucosal sites, is one of the possible causes of this phenomenon.

Detection of PF can have several implications in clinical practice. As demonstrated by Lodi et al. [29], it is possible to avoid a prophylactic antifungal treatment in those patients undergone a local corticosteroid therapy, provided that they demonstrate a negative *Candida* carriage: on the basis of the negative predictive value of autofluorescence that we found, the PF finding could allow the identification of patients eligible to receive a preventive antifungal treatment. Moreover, the topographic distribution of PF can permit a selective swab in specific oral mucosal sites, representing a fast, non-invasive and relatively inexpensive method for an initial chair-side screening for the *Candida* detection. A future development of this pioneering study could consist in the enrolment of high-risk groups (e.g. diabetics, immunosuppressed, removable oral prosthesis wearers) or patients with a confirmed diagnosis of oral candidiasis or subjects with co-existing red/orange and green areas on the tongue.

Further investigations are needed to understand how the presence of PF is related to the absence of *Candida*. Our hypothesis is that specific cluster of bacteria responsible for PF antagonize establishment and growth of *Candida* species. This can be owed to an interspecies competition for the colonization of the dorsal tongue ecological niches: a similar mechanism has already been demonstrated between *Candida* and Pseudomonas aeruginosa [30]. Another possible explanation is an antifungal activity of porphyrin itself [31, 32].

Further studies could better identify these porphyrin bacteria species and their metabolic products. The close link between PF and oral bacteria has been recently proven by Liu et al., that demonstrated the efficacy of red fluorescence imaging as an objective and promising method for dental plaque detection and quantification [33].

However, PF detection thorough VELscope does have limitations: it is not possible to define the *Candida* species involved in the colonization, hence PF should be considered only as an initial and complementary screening aid. Moreover, in the present study we did not perform a culture test to identify the bacteria species associated to PF, representing an implication for future researches. This method could also be implemented with a computerized image analysis, using dedicated software for standardized colours identification and evaluation.

Further randomized controlled trials are needed to test the effectiveness of presence/absence of PF in *Candida* detection. The "off-label" use of autofluorescence proposed in this study has evidenced the potential of light technologies as screening support for oral diseases.
